# Flexible Triboelectric Mechanical Energy Harvesters for Wearable and Self-Powered Sensing Applications: A Review

**DOI:** 10.3390/s26041166

**Published:** 2026-02-11

**Authors:** Manchi Punnarao, Hong-Joon Yoon

**Affiliations:** 1 Department of Semiconductor Engineering, Gachon University, Seongnam 13120, Republic of Korea; 2 Department of Electronic Engineering, Gachon University, Seongnam 13120, Republic of Korea

**Keywords:** polymer/composite films, mechanical energy harvesting, wearable electronics, self-powered sensors, TENGs

## Abstract

**Triboelectric** nanogenerators (TENGs) have been gaining significant attention owing to their excellent energy conversion efficiency and their integration towards a large number of practical applications in energy harvesting, wearables, and self-powered sensing. In recent advancements, the utilization of flexible triboelectric composite films can help to enhance the TENG’s electrical output performance, as they possess excellent mechanical and dielectric properties and tunable surface characteristics. Moreover, by combining flexible active layers with triboelectric nanogenerators, the advantages of each component result in sensor devices which offer superior characteristics, including high sensitivity, biocompatibility, less weight, and mechanical flexibility. This review mainly focuses on the applications of TENGs in mechanical energy harvesting, self-powered wearable sensor systems, as well as the latest research progress in the TENG field. The working principles of TENG will be first explained in detail, including four basic operational modes of TENG, simulation results, and the working mechanism of the contact–separation mode TENGs. The fabrication techniques of triboelectric flexible films, along with TENG construction, will then be introduced. Common applications of TENGs are based on mechanical energy harvesting and powering portable electronic devices, which will subsequently be classified and summarized. Additionally, the applications of various wearable and self-powered sensor applications are elucidated. Finally, the current limitations and future directions of the TENG will be explained in detail and proposed. By exploring these innovations, the review underscores the importance of triboelectric flexible film-based TENGs in driving the future of energy harvesting and sensor technologies.

## 1. Introduction

With the rapid expansion of smart, flexible, and wearable electronic devices and the growing energy crisis caused by the reduction in fossil fuels, mechanical energy harvesting from the living environment to power wearable and low-powered portable electronics has attracted increasing attention [1,2]. In parallel, the widespread evolution of portable and wearable electronics has raised the demand for continuous, dependable energy sources. Although modern batteries offer improved energy density and longer operating times, their fundamental limitations, such as storage capacity, weight, and size, and the continuous need for recharging, still pose significant challenges [3,4]. These limitations are particularly restrictive for mobile and remote applications that support the expansion of the Internet of Things [5]. The development of green energy harvesting technologies currently plays a key role in addressing worldwide energy demands and environmental interests. Different types of energy harvesting technologies exist to convert different external energy sources, such as mechanical, wind, tidal, sound, and thermal energy, into electricity [6,7,8]. Examples of energy harvesting mechanisms in such technologies include the utilization of piezoelectric, triboelectric, thermoelectric, and electromagnetic effects.

Among various harvesting technologies, triboelectric nanogenerators (TENGs) are one of the promising techniques that convert mechanical energy into electricity, which is vital for self-powered systems. In the year 2012, Z. L. Wang and his co-workers proposed a TENG based on the contact electrification and electrostatic induction phenomena [9]. TENGs have attracted more interest due to their advantages, such as simple design, high energy conversion efficiency, lower cost, long-lasting nature, and a wide range of possible materials used in TENG fabrication [10,11,12]. For example, H. Luo et al. proposed a multi-phase rotating disk TENG (MPRD-TENG) with excellent DC electrical output for operating fire warning alarm systems and speed-monitoring applications [13]. Moreover, H. Luo et al. designed a highly stable rotary solid–liquid TENG (RSL-TENG) for wastewater detection applications, which are abundantly available in temperature, humidity, and light-sensitive monitoring modules [14]. Since the concept was invented, TENGs have been extensively investigated in various application areas, such as energy harvesting, healthcare monitoring, robotics, and sensor devices [15,16,17,18]. Also, several methodologies have been improving the TENG output power, such as structural design, charge injection, and material optimization by incorporating fillers, etc. [19,20,21,22]. So far, several organic polymers and inorganic materials, dielectrics, carbon materials, filler materials, and loaded polymers (composites) have been selected for designing flexible and high-output TENGs, such as BaTiO_3_, KNaNbO_3_, Mxene, CNTs, PVA, PVDF, PDMS, etc. [23,24,25,26,27]. Triboelectric charges are built on surfaces where dissimilar materials with opposite electron affinities contact by external friction. The polymers and composite films improve the surface charge density, mechanical strength, and electrical properties of materials, resulting in high electrical output from the corresponding TENG. Also, their essential beneficial factors include easy incorporation, biocompatibility, and high energy-conversion efficiency, which make TENGs highly promising for self-powered sensing and wearable applications [28,29,30]. Such devices can employ various forms of mechanical energies, like pressure, stretching, vibration, and rotations, and convert them into electrical energy. The compatibility of polymer and composite polymer film-based materials allows for high flexibility, lower cost, high mechanical strength, and easy fabrication, making them ideal for next-generation applications.

**Figure 1:**
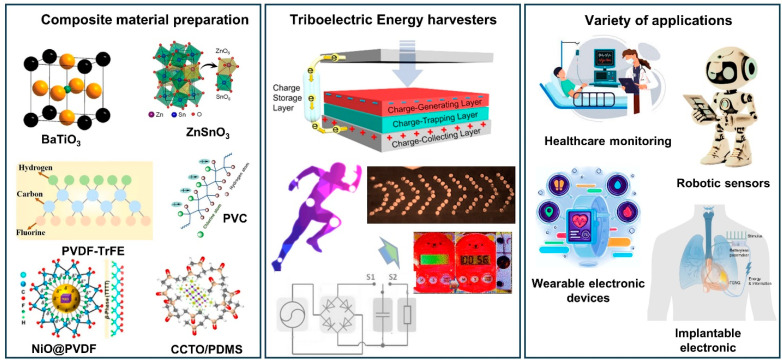
Overview of flexible TENG. Fabrication of organic, inorganic, and composite-based TENGs for energy harvesting and a variety of smart sensing applications [31,32,33,34,35,36,37,38,39,40]. All essential copyrights and permissions received.

This review highlights recent progress in the area of polymer/composite films employed in flexible TENGs and their smart applications. The discussion includes operation modes, working principles of TENGs, and the mechanism of the contact–separation mode of TENGs was explained in detail. Also, the COMSOL multi-physics simulation results are explained. Further, various classes of polymer/composite films and TENG fabrication techniques are systematically analyzed to explain how their design structures contribute to charge-generation efficiency, mechanical properties, and interfacial stability. The capability of these engineered film-based TENGs delivers high output performance while maintaining flexibility and long-term operational stability, making them promising candidates for next-generation smart electronics. Therefore, the practical and real-time applications of the TENG under mechanical energy harvesting, wearable, and smart self-powered sensors were systematically investigated. However, significant challenges endure, such as optimizing filler materials in polymer matrix interfaces, confirming stable electric output under constant mechanical loading, and developing eco-friendly fabrication methods. Overcoming these limitations is vital for the development of flexible TENGs constructed from organic, inorganic, and hybrid composite films, thereby supporting their reliable use in advanced wearable sensing devices, soft robotic systems, and health-monitoring applications, as shown in Figure 1, refs [31,32,33,34,35,36,37,38,39,40].

## 2. TENG Working Principle

Self-powered electronic applications utilizing the triboelectric effect show significant capabilities in converting mechanical energies into electrical power, eliminating reliance on external power sources. These devices boast increased consistency, scalability, and compactness, offering unparalleled versatility in a variety of applications. In this section, starting from the triboelectric working principle, the operational working modes of various TENGs will be described, with a detailed emphasis on the working mechanism of the contact–separation-mode TENG. The triboelectric effect occurs abundantly in living environments and generates electrical charges through mechanical actions, such as contact and separation between a pair of materials. Prof. Z. L. Wang and his group devised the TENG in 2012 by combining the effects of contact electrification and electrostatic induction, thus providing a new direction through the development of a novel energy source for the era of green energy [9].

**Figure 2:**
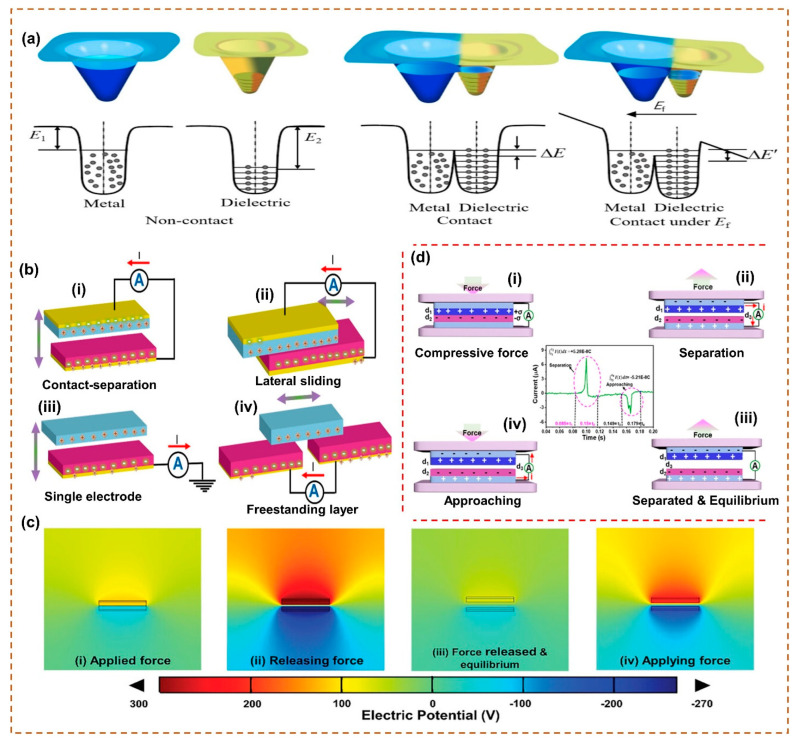
(**a**) Working principle of TENG [44]. (**b**) Different types of operational modes of TENG: (**i**) contact–separation mode, (**ii**) freestanding mode, (**iii**) sliding mode, and (**iv**) single-electrode mode [47]. (**c**) (**i**–**iv**) COMSOL Multiphysics simulation results of TENG [58]. (**d**) (**i**–**iv**) Working mechanism of the CS-mode TENG [59]. All essential copyrights and permissions received.

The principles of charge flow between the two triboelectric material surfaces have been extensively reviewed and can generally be split into three types: electron, ion, and material transfers [41,42,43]. Electron transfer is widely considered the main charge transfer process in solid–solid triboelectrification, particularly for insulating and semiconducting materials. When two surfaces come into close contact, their surface electronic states interact, causing electrons to move from the material with a higher Fermi level to the material with a higher work function [41]. Ion transfer becomes important in triboelectric processes that involve liquids, soft materials, or humid conditions [42]. Material transfer happens when tiny molecular parts, polymer chains, or nanoscale particles are physically shifted from one surface to another during contact. This effect is most often seen in soft polymer materials, elastomers, and rough surfaces [43]. Together, these mechanisms determine the surface charge and electric potential that enable energy generation in triboelectric nanogenerators. For example, G. Xu et al. proposed that electron transfer is the main process of contact electrification and put forward an electron cloud model to explain the relevant mechanism [44]. Figure 2a describes, on an atomic level, the electronic behavior when two originally separated layers, each containing a triboelectrically active material, come into contact. When separated, the electrons in each layer are strongly bound within their respective potential wells and specific orbitals as they are within the attractive region of their respective atomic potential. Their corresponding electron clouds, therefore, remain separated without overlapping, and charge escape and transfer are barred. When the atoms of the separated layers approach and come into contact, their respective electron clouds overlap, and charge “screening” lowers the energy barrier between the two materials, allowing the originally distinct potential wells of each layer to merge and transform into an asymmetric double-well potential. The chemical potentials of the surfaces of the layers also equilibrate during contact, facilitating electron transfer. When the layers are separated, if the rate of mechanical separation exceeds the rate of electron re-equilibration, then the transferred electrons would remain on the material surface as static charges, causing both triboelectric materials to exhibit positive and negative polarities, respectively.

## 3. Operational Modes of TENG

Depending on the structural design and the shape of the triboelectric materials in a TENG device, four types of fundamental operational modes have been defined, such as contact–separation TENG (CS-TENG), single-electrode (SE-TENG), lateral-sliding (LS-TENG), and freestanding (FS-TENG) modes, as shown in Figure 2b, refs [45,46,47]. According to different electrode configurations, it can be split into two types, i.e., single and double electrode types.

The most common and simple fundamental form of operation mode is the CS-mode TENG, as shown in Figure 2b(i), which is applied when the relative motion is perpendicular to the interface [48,49,50]. The two dissimilar dielectric materials are oppositely placed, and the electrodes are attached to each dielectric material. When the two dielectric materials come into contact by applying friction, a charge is generated on the active materials. When both dielectric layers are separated by removing friction, the surfaces of both dielectric layers preserve their electric charges, which can lead to a potential difference between the two dielectric materials. As a result, an electrostatic field is generated, and a charge transport starts from one electrode to another. When the dielectric materials come into contact again, the electrostatic field vanishes, and electron flows return. The result of the alternating current signal will be generated by this continuous contact–separation TENG process. CS-TENG provides a fundamental and effective route for converting mechanical energy, characterized by simple design, stable electrical output performance, and wide adaptability to mechanical stimuli. Advances in materials, structural design, and device integration continue to enhance their performance and support their application in self-powered and sustainable electronic systems.

In the lateral-sliding-mode TENG (LS-TENG), the two different dielectric materials remain in direct contact, and a frictional force induces sliding or rotational motion, thus leading the effective contact area to change constantly, as shown in Figure 2b(ii) [51,52,53]. This interaction generates triboelectric charges on the dielectric material surfaces, leading to a changing potential difference between the electrodes and generating an alternating output current. Compared with the CS mode, the LS mode configuration facilitates more effective charge generation, supplies higher electrical output, and does not require an air gap for surface separation. Nevertheless, LS mode is limited by the intense friction that occurs during operation, which can accelerate material degradation and reduce the overall lifespan of the TENG. LS-TENGs offer an efficient route for harvesting mechanical energy, supported by continuing developments in device architecture, surface modification, and power regulation. Also, continued interdisciplinary efforts are expected to improve their reliability and facilitate their integration into self-powered and sustainable electronic systems.

In the single-electrode-mode TENG (SE-TENG) (Figure 2b(iii)), a single grounded electrode, typically composed of a material with less affinity to attract electrons, functions as both the reference electrode and the triboelectric dielectric layer [54,55]. This electrode interacts with a freely moving dielectric layer that has a higher electron affinity. When the dielectric material approaches the grounded electrode, electrostatic induction drives electrons from the electrode toward the ground. As the dielectric material goes away, the induced potential reverses, causing electrons to go back from the ground to the electrode. Although it has a simple and versatile nature, the SE mode frequently suffers from limited electron-transfer efficiency, which reduces the overall power output. SE-TENGs offer a simple yet adaptable solution for mechanical energy harvesting and self-powered sensing. Continuing advances in triboelectric materials, device fabrication, and system integration are expected to enhance their output power and accelerate their adoption in wearable electronics and delivered sensing systems.

In the freestanding-mode TENG (FS-TENG), two symmetrical electrodes connected through an external circuit are positioned under a movable dielectric triboelectric material, as shown in Figure 2b(iv) [56,57]. When the dielectric material shifts relative to the electrodes, the spatial distribution of electrostatic potential is altered, driving electrons to move back and forth between the electrodes and generating an electrical output. Because charge transport occurs through electrostatic induction rather than direct mechanical rubbing between solid surfaces, this mode significantly reduces surface wear and improves the long-term durability of the TENG. FS-TENG efficiently harvests mechanical energy by coupling the moving triboelectric layer to the fixed electrodes, which minimizes mechanical wear and supports varied motion modes. This design enhances electrostatic charge induction, while continued advances in triboelectric layers, device structures, and system integration are expected to elevate performance further and expand applications in self-powered electronics, smart sensing, and sustainable energy harvesting.

In addition, the working mechanism of the CS-mode TENG was further investigated using COMSOL simulation results to show the obtained electric potential during the contact and separation operation. Based on the observed surface charge density and dielectric constant of the triboelectric materials, the generated electric potential from the TENG was observed in different stages by performing an analysis using a finite element analyzer, solver, and COMSOL simulation software. The detailed device simulation results are provided in Figure 2c(i–iv), ref. [58]. The highest electric potential was produced when the TENG was in approaching and separating stages, and the minimum potential was produced when the device was in idle, contact, and separated stages.

## 4. Working Mechanism of CS-Mode TENG

The working mechanism of the vertical contact–separation-mode TENG was explained in detail, and it depends on the coupling effects of triboelectrification and electrostatic induction, as shown in Figure 2d, refs [48,49,50,59]. This CS mode was mainly divided into four stages. First, both the dielectric layers fully contact by the friction applied on the TENG (Figure 2d(i)), and the positive and negative charges are produced on the surface of the dielectric materials. The produced charges do not transfer and are quickly neutralized owing to the insulating nature of the dielectric layers. When the dielectric materials start to separate due to the removal of friction on TENG (Figure 2d(ii)), the electric potential difference is created in both electrodes, leading to electron flow transfer from one electrode to another. As a result, the triboelectric positive electric output is generated from the TENG. When the dielectric material is completely separated or reaches an equilibrium state by removing friction on TENG (Figure 2d(iii)), the potential difference is completely offset by the electron flow between the electrodes, and no electric potential is produced in the external circuit. Furthermore, both dielectric materials make contact again by applying friction on TENG (Figure 2d(iv)), creating a reverse electrical output signal via an external circuit because of the backflow of the electrons. Consequently, continuous external friction applied and removed on the TENG could produce a continuous alternative output current signal.

## 5. Fabrication Techniques of Triboelectric Flexible Films Along with TENG Design

**Figure 3:**
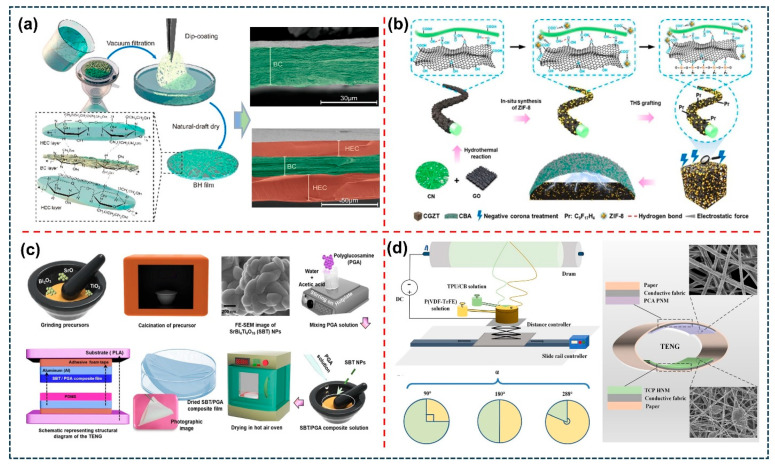
Fabrication techniques of polymer/composite films based on TENG. (**a**) Preparation process of bacterial cellulose fiber-based TENG device, which is fabricated using vacuum-filtration and dip-coating techniques [64]. (**b**) Schematic representation of ZIF-8 decorated carboxycellulose nanofiber/graphene oxide aerogels (CG aerogels) via a hydrothermal reaction for the construction of flexible TENG [65]. (**c**) Schematic diagrams and fabrication of SBT NPs loaded with PGS composite films via the solid-state synthesis technique for TENG design [59]. (**d**) MFSE preparation process of TCP HNMs with P(VDF-TrFE), two adjustable regions, and assembled TENG [68]. All essential copyrights and permissions received.

Various effective materials synthesis techniques have been implemented to design high-performance TENG devices, along with flexible triboelectric films, addressing diverse energy harvesting and various self-powered sensing applications, as shown in Figure 3. These techniques include hydrothermal, vacuum filtration, plasma treating, tape casting, spin and dip coating, doctor blade, 3D printing, spray coating, electrospinning, etc., to prepare flexible triboelectric films and design a TENG. In this case, to prepare flexible triboelectric films and high-performance TENGs, various materials were selected, such as polymer materials, dielectric/ferroelectric materials, conductive fillers, and combined filler materials in polymers for forming composite films. For example, A. Gupta. et al. proposed a novel metal-coupled NaYF_4_ dielectric polymer matrix, which is prepared by an in situ hydrothermal process and doped with lanthanide ions (NaYF4: Yb, Er@Ag nanocomposites, NCs) mixed in Ecoflex and polyester cloth (PC) [60]. Further, the TENG is designed with and without a polymer spacer, and both triboelectric materials act as triboelectric positive and negative layers. The fabricated TENG exhibits excellent electrical output performance and harvests mechanical energy from human activities, as well as further utilizes it for wearable applications. W. Wang et al. prepared a Pd@ZnO/MoSe_2_ nanoflower via a solvothermal synthesis reaction, and further, the Pd@ZnO/MoSe_2_ solution was decorated on a Cu-Ni electrode using a spray-coating technique for the gas sensing and TENG applications [61]. E. Cho. et al. developed a hierarchical-wrinkled-architecture TENG (HWA-TENG) based on the plasma–polymer–fluorocarbon (PPFC) thin film [62]. The fabricated thin film is employed as a high-performance triboelectrification layer due to the high surface charge potential, water repellency, transparency, and ultrathin thickness. This proposed PPFC-based HWA-TENG demonstrated excellent electrical output and was successfully employed as a raindrop energy harvester. C.K. Chung et al. developed a micro-needle structure created on a PDMS/conductive polyester fabric-based MN-TENG device [63]. In this study, microstructures were designed using laser engraving machines. The fabricated MN-TENG exhibits a stable, high electrical output and is used to power various low-powered electronic devices. Also, C. Luo. et al. established an eco-friendly and recyclable bacterial cellulose-based flexible triboelectric nanogenerator (BC-TENG), as shown in Figure 3a, ref. [64]. Here, the surface potential of BC films was controlled and designed using a dip-coating hydroxyethyl cellulose solution to improve the electrical output performance of the BC-TENG. In addition, the BC-TENG exhibits outstanding electrical output stability and is effectively employed as a self-powered sensor to detect the mechanical movements in daily human life and power portable electronic gadgets. As shown in Figure 3b, F. Wang. et al. developed a TENG self-powered filter using triboelectric material ZIF-8 decorated on the surface of cellulose/graphene oxide aerogel (CN/GO aerogel) and grafting it with trimethoxy(1*H*,1*H*,2*H*,2*H*-heptadecafluorodecyl) silane using negative corona treatment [65]. The fabricated TENG generated excellent output performance via electrical energy converted from the mechanical energy through the breathing-driven mechanism. From these results, the TENG is utilized for different applications such as wearable and self-powered healthcare monitoring. Moreover, A. Kurakula et al. prepared a strontium bismuth titanate nanoparticle (SBT NPs) incorporated polyglucosamine (PGA) (SBT/PGA) composite film-based flexible TENG, as shown in Figure 3c, ref. [59]. The SBT NPs were synthesized via a solid-state synthesis technique. The prepared composite films show excellent dielectric properties, which can lead to improvement in the electrical performance of the corresponding TENG. S. He et al. established a TENG device based on the porous/patterned surface PE/PDMS and PA/PEG triboelectric films, which are prepared via a traditional 3D printing technique [66]. The fabricated TENG shows excellent flexibility, a short fabrication cycle, lower cost, and the possibility of large-area modeling properties, along with high electrical output. These results can stimulate the industrialization of the proposed TENG and make it more accessible for practical applications. Furthermore, B. Amrutha et al. prepared a copper oxide nanoparticle (CuO NPs) loaded with PVDF composite nanofibers—a TENG device for energy harvesting applications [67]. The CuO/PVDF composite nanofibers were fabricated via the conventional electrospinning technique. The proposed TENG exhibits excellent electrical output and harvests energy from daily human activities. In addition, J. Yin et al. have developed a flexible TENG based on hybrid-structured nanofiber membranes (HNMs) using TPU/CB beaded nanofibers and PVDF-TrFE nanofibers by a modified free-surface electrospinning technique (MFSE), as shown in Figure 3d, ref. [68]. The fabricated HNM-based TENG exhibited superior electrical output and was further employed as a mechanical-energy harvesting device to convert biomechanical energy from daily human activities. M. Kim et al. developed a high-performance TENG using a composite film of PEDOT:PSS-loaded PVDF-TrFE and MAPbI_3_ particles [69]. The composite film was fabricated using a spin-coating technique. The fabricated perovskite thin film-based TENG exhibited an excellent electrical output, and the TENG was employed to harvest mechanical energy for powering various electronic devices. Moreover, J. Mao et al., H. Zhu et al., H. Patnam et al., P.S. Das et al., Y. Lui et al., and T. Huang et al. also designed a flexible polymer/composite film via different fabrication techniques, such as blade coating, casting/rolling, solution casting, vacuum filtration, spin coating, and electrospinning [70,71,72,73,74,75]. The prepared flexible films were utilized to fabricate TENG devices for energy harvesting and sensor applications. From these results, flexible TENGs are fabricated based on the flexible polymer/composite films via different techniques, which are easy, scalable, lightweight, budget-friendly, and environmentally friendly. Therefore, the flexible triboelectric polymer or composite films based on TENG have unique advantages in the various energy harvesting, wearable electronic, and self-powered sensor fields. A summary of a comprehensive list of different fabrication processes of polymer/composite film-based TENGs for various TENG applications is shown in Table 1. As material technologies continue to progress, constructing highly flexible triboelectric polymer and composite films will gradually become more important for enabling more efficient and flexible TENG constructions.

## 6. Applications of Flexible Polymer/Composite Films Based on TENG

The combination of TENGs designed from flexible polymer/composite films into specific applications offers significant potential to enhance their electrical output and large-scale practical applications. The films enable efficient energy conversion and open a wide range of applications owing to their adaptable structural properties. This combination plays a key role in the advancement of self-powered sensing devices and holds great promise for human biomechanical energy harvesting, wearable, and self-powered smart sensing applications.

## 7. Mechanical Energy Harvesting

**Figure 4:**
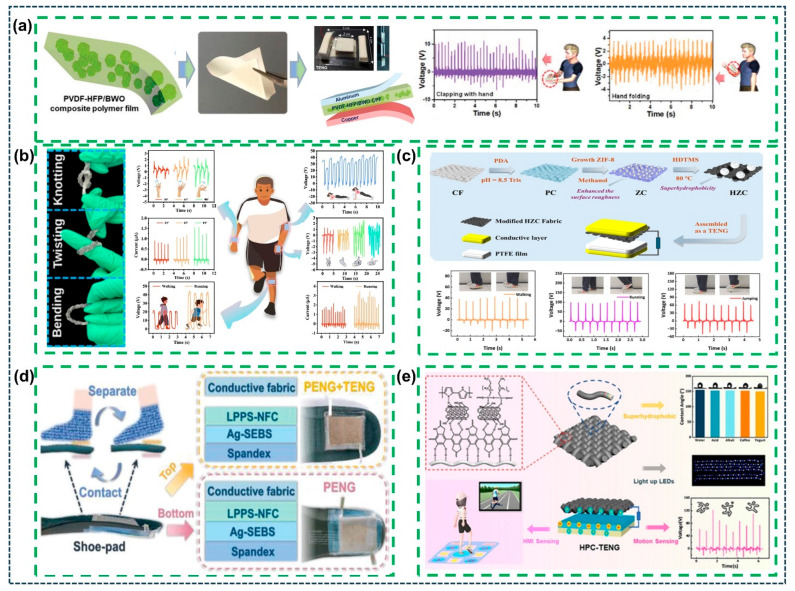
Practical applications of the TENG for mechanical energy harvesting. (**a**) Schematic illustration of the PVDF-HFP/BWO composite polymer film-based TENG for mechanical energy harvesting [76]. (**b**) Photographic images of the coaxial optical fiber self-powered system and energy harvesting from human body activities [78]. (**c**) Construction process of HZC fabric-based TENG device and its real-time mechanical energy harvesting applications [83]. (**d**) Schematic illustration of the LPPS-NFC-based flexible and wearable TENG device placed on the shoe pad actuated by the foot stamping for energy harvesting and sensing applications [84]. (**e**) Schematic diagram representation and output results of HPC fabric-based TENG for mechanical energy harvesting, wearable, and smart sensing applications [85]. All essential copyrights and permissions received.

Within the last couple of decades, several studies have been conducted on the flexible polymer/composite film-based TENGs for biomechanical energy harvesting, wearable, and self-powered sensing applications. P. Manchi et al. constructed a high-performance flexible TENG device based on the dielectric and ferroelectric bismuth tungstate (Bi_2_WO_6_) marigold flower-like structures by hydrothermal synthesis and further embedded them into a poly (vinylidene fluoride-co-hexafluoropropylene) (PVDF-HFP) polymer to form a flexible PVDF-HFP/Bi_2_WO_6_ composite polymer film (CPF), as shown in Figure 4a, ref. [76]. The fabricated flexible PVDF-HFP/Bi_2_WO_6_ CPF exhibited a high polar crystalline β-phase, a high piezoelectric coefficient, and ferroelectric properties, which can improve the TENG’s electrical output. The proposed TENG shows a highly stable and durable electrical output over the long term, harvesting mechanical energy from everyday human body activities and powering various portable electronic devices. Also, Y.L. Yashaswini et al. designed high-performance TENGs using functionalized reduced graphene oxide (FrGO) in the polyvinyl alcohol (PVA) nanocomposite [77]. The fabricated flexible FrGO nanocomposite-based TENG exhibited an electrical output 17 times higher, as compared to pristine PVA-TENG. Further, the proposed TENG demonstrated a practical applicability, demonstrating its ability to power LEDs and digital timers via charging commercial capacitors using bridge rectifiers. Q. Ju et al. proposed a reduced graphene oxide (rGO) and polyaniline (PANI) decorated on a carbon fiber (CF) core electrode-based TENG, as shown in Figure 4b, ref. [78]. The fabricated TENG exhibits excellent electrical output and was also utilized for energy storage applications using a hybrid self-powered supercapacitor. The proposed model utilizes mechanical energy from daily human activities for harvesting and employs it to power electronic devices. Also, R. Umapathi et al. proposed lead-free strontium-doped bismuth ferrite (BSFO) nanoparticles with multiferroic and dielectric behavior via a solid-state synthesis technique and further incorporated them into PDMS to fabricate the BSFO/PDMS composite film-based TENG [79]. The fabricated BSFO-TENG delivered excellent power density and long-term operational stability. Furthermore, it illuminated various commercial LEDs, powered a digital clock, harvested electrical energy from daily human body motions, and was utilized for security alert applications. M. Mao et al. developed a biodegradable, recyclable, and biocompatible composite film-based TENG using a polyethyleneimine (PEI)/iron chloride (FeCl_3_)/microcrystalline cellulose (MCC) composite film [80]. The fabricated MCC/PEI/FeCl_3_ composite films based on TENG demonstrated exceptional pressure sensitivity and high-humidity sensing, fatigue resistance, and highly stable and long-term durable electrical output. Furthermore, the device exhibits high sensitivity to human body activities, such as walking, running, jumping, coughing, and breathing, etc. From these results, the proposed TENG is employed to harvest mechanical energy from the daily human actions and pressure-sensing applications. In addition, V. Mohan et al. fabricated a TENG using sonochemically prepared graphene/polydimethylsiloxane (SGP) nanocomposite and PEO layers [81]. The proposed TENG shows excellent electrical output, and the device can be successfully combined into practical applications such as human motion monitoring in daily activities, gaming interfaces, and power-point control applications. In a more fundamental study, C.M. Veerabhadraswamy et al. proposed a natural fiber-based TENG comprising PVA, and the agro-waste composites were prepared using a solution casting method [82]. The fabricated TENG exhibited high electrical output and powered various low-powered portable electronics, including LEDs and LCD timers. Also, it functioned biomechanical sensor for harvesting mechanical energy from daily human motions. M. Qu et al. established a flexible superhydrophobic MOF-based textile TENG for mechanical energy harvesting and wearable electronic applications, as shown in Figure 4c [83]. The fabricated TENG shows high electrical output and high durability even after long-term study over several weeks. The TENG demonstrated mechanical harvesting from human body activities and powering electronics, as well as self-powered sensors in human motion detection and Morse code information transmission. Furthermore, the conversion of mechanical energy into electrical energy from human mechanical movements by operating a TENG can be thought of as a sensing capability to be used in wearable applications. This functionality can be observed as high responsivity to voltage and current variations within a sensible time. In this case, flexible TENGs can be a perfect wearable sensor, with a quick response and sensitivity to mechanical stimuli. F. Jiang et al., in Figure 4d, developed a breathable, stretchable, and stable nanofiber composite (LPPS-NFC) via the electrospinning technique based on the Cs_3_Bi_2_Br_9_ perovskites decorated in lead-free perovskite/poly (vinylidene fluoride-co-hexafluoropropylene) (PVDF-HFP) and styrene-ethylene-butylene-styrene (SEBS) [84]. The LPPS-NFC-based TENG demonstrated excellent electrical output, enabling it to show a robust wearable device that converts mechanical energy from the different mechanical motions into electricity to operate various portable electronics. These results demonstrate that the proposed LPPS-NFC-based TENG can endure extreme deformation while maintaining excellent electrical output, highlighting its strong potential for use in smart textiles and wearable power sources. Moreover, J. He et al. presented a novel superhydrophobic and conductive composite fabric (HPC) using polydopamine (PDA), carbon nanotubes (CNTs), and polypyrrole (PPy), which were loaded on the cotton fabric surface to create the hydrophobic nature of cetyltrimethoxysilane (HDTMS), as shown in Figure 4e, ref. [85]. The prepared HPC exhibits excellent wear resistance, breathability, and self-cleaning properties, as well as high and stable electrical output from the HPC-TENG. The proposed TENG demonstrated mechanical energy harvesting from human activities and powering various electronics, as well as utilizing human–machine interface sensors for gaming blankets.

## 8. Self-Powered Sensors

**Figure 5:**
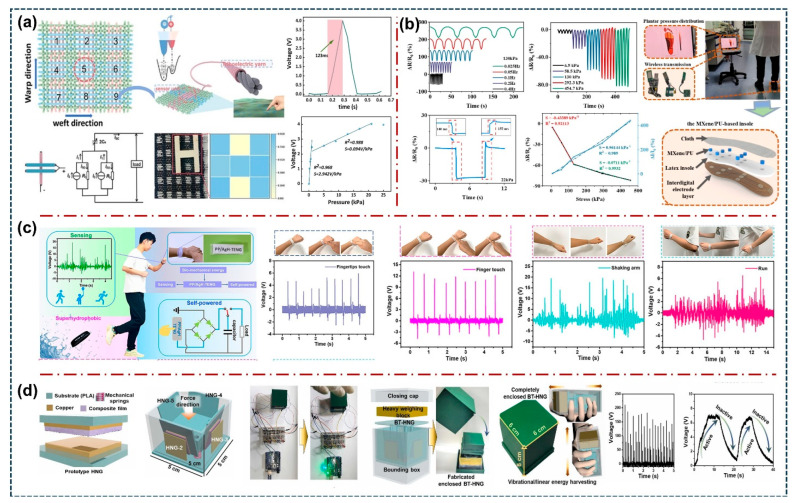
(**a**) Schematic diagram illustration and fabrication of triboelectric fabric sensor array (TFSA) for pressure sensor applications [86]. (**b**) 3D microporous MXene/polyurethane (MXene/PU) composite gels based on ultra-high stable pressure sensors and flexible triboelectric nanogenerators for gait monitoring applications [90]. (**c**) Core-shell superhydrophobic and flexible triboelectric nanogenerator based on the PDMS film surface modified with PTFE particles as the triboelectric layer and AgNWs/PVA hydrogel electrodes for biomechanical energy harvesting and wearable self-powered sensing applications [91]. (**d**) Schematic representing the structural composition of the fabricated HNG, along with the heavy-weighting block enclosed in a 3D-printed box for vibrational/linear mechanical energy harvesting applications [93]. All essential copyrights and permissions received.

In addition to detecting mechanical stimuli, physiological signals, and pressure-sensing parameters, increasing research efforts are being directed toward monitoring a wide range of environmentally relevant factors, reflecting the rising attention to sustainable and eco-friendly sensing technologies. For example, Q. Tao et al. proposed a triboelectric fabric pressure mapping sensor with high sensitivity and free crosstalk using polytetrafluoroethylene/stainless steel (PTFE-SS) braided yarns and the polyamide 66/stainless steel (PA66-SS) braided yarns in the weave fabric, as shown in Figure 5a, ref. [86]. The fabricated fabric-based pressure sensor TENG demonstrated excellent sensitivity of 2.942 V·kPa^−1^ and a fast response time of 123 ms. This proposed device was also utilized as a sensor for human motion sensing while doing daily activities, as well as a touch sensor. T. Yang et al. proposed sheath-core PVDF/graphene/carbon fiber (PVDF/G-CF) yarns fabricated using a conjugate electrospinning technique, covering a commercial CF core and an electrospun graphene-doped PVDF sheath [87], which can enhance the fatigue resistance of electrospun nanofibers under prolonged friction and keep a high degree of freedom. The resulting electronic textile, woven with large-area electrospun PVDF/G-CF yarns, delivers a high-power density of 25.5 mW/m^2^. The uniform distribution of PVDF/G nanofibers on the textile surface provides good softness, washability, and long-term durability. Further, the proposed electrospun PVDF/G-CF textile shows strong potential for pressure sensing, motion monitoring, and self-powered operation, making it a strong potential application for next-generation wearable electronic systems. X. Wang et al. proposed a soft and stretchable TENG skin for energy harvesting to operate portable electronics from biomechanical energy [88]. The fabricated TENG exhibits excellent electrical output and has desirable features like biocompatibility, simple fabrication, lightweight, and environmentally friendly nature. The TENG is integrated with a power management unit to drive electronic devices by harvesting energy from hand motion. Also, the proposed TENG provides a new insight for clean power sources of skin-mounted electronic gadgets and promotes the development of sustainable energy sources for wearable and portable electronics. J. Huang et al. established a novel self-powered pressure sensor based on the bacterial cellulose/chitosan (BC/CS) composites and copper nanoparticles-doped polydimethylsiloxane (PDMS/CU) films [89]. The fabricated device demonstrated excellent mechanical stability and high-pressure sensitivity of 0.24 V/kPa^−1^. The device exhibits high sensitivity when it is attached to human body parts, and it detects joint motions. In addition, S. Wang et al. developed 3D microporous MXene/polyurethane (MXene/PU) composite gel-based triboelectric nanogenerators, as shown in Figure 5b, ref. [90]. The fabricated Mxene/PU exhibits exceptional performance characteristics such as a large compressive range, excellent cycling durability, long-term stability, and high sensitivity of 0.96144 kPa^–1^. The proposed TENG generates excellent electrical output performance as well as remarkable effectiveness in applications of healthcare monitoring and rehabilitation. M. Qu et al. fabricated a superhydrophobic, humidity-resistant flexible TENG based on the PDMS/PTFE and AgNWs/PVA hydrogel electrodes, as shown in Figure 5c, ref. [91]. The fabricated PP/AgH-TENG generated a high electrical power density of 3.07 W/m^2^ and can easily power several portable electronic devices. Additionally, the bracelet-type TENG was developed to harvest mechanical energy from human movements, as well as demonstrated its potential in the field of wearable sensing applications. Also, Y. Liu et al. have developed a rapid-response, highly sensitive, and self-powered artificial sensory memory actuated by a triboelectric sensory receptor (TESR) for real-time neuromorphic computing applications [92]. The flexible TESR is designed based on the DPP-DTT and ion-gel layers. The device exhibits high electrical output and pressure-sensing properties with a sensitivity of 0.192 kPa^−1^. Further, several TESR devices were arranged in a matrix shape to demonstrate handwritten image recognition and neuromorphic computing results. These results from TESR sensors have strong potential for real-time broad applications in neuromorphic systems, human–machine interaction, and large-scale neural networks. Also, M.V. Paranjape et al. have proposed a 3D-printed box-type hybrid nanogenerator (BT-HNG) based on the acetone-dissolved microarchitectured strontium (Sr)-doped silver niobate (ASNb)/ecoflex composite film for efficient mechanical energy harvesting and sensor applications (Figure 5d), ref. [93]. The highly effective and stable electrical output exhibited by BT-HNG is further utilized for powering small-scale electronic devices. Moreover, the proposed BT-HNG successfully demonstrated its use as a real-time sensor that can be utilized for powering the stairs, corridors, streetlamps, etc., in human presence, as well as harvesting vibrational/linear movements into electrical energy.

**Figure 6:**
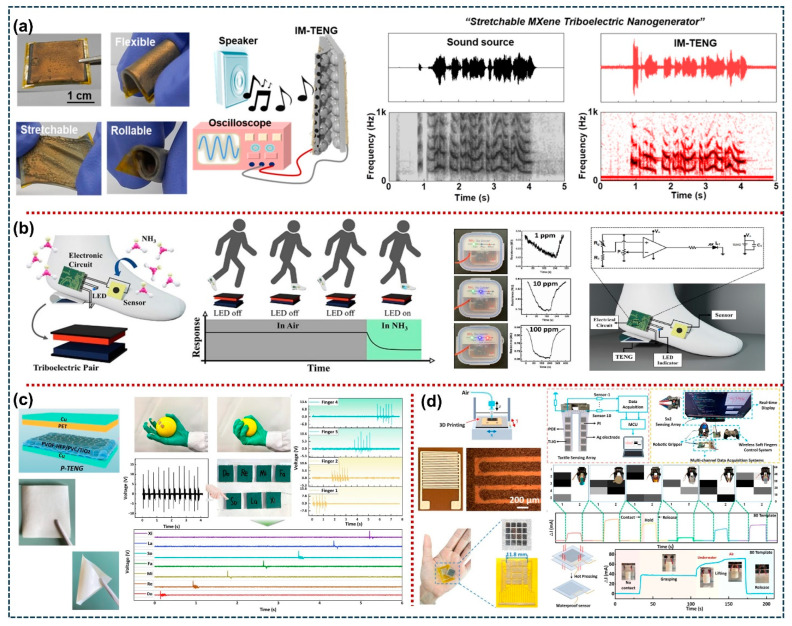
(**a**) Photographic images of the IM-TENG determining its flexible, stretchable, and rollable nature, further the experimental setup and output results of the acoustic wave sensing [95]. (**b**) Schematic representation of the MXene/TiO_2_/cellulose nanofiber-based TENG integrated shoe for energy harvesting and self-powered gas sensing applications [96]. (**c**) Schematic diagram and photographic images of flexible PVDF–HFP/PVC/TiO_2_ composite film-based TENG for environmental energy harvesting and self-powered tactile sensing applications [100]. (**d**) Templated laser-induced graphene (TLIG)-based 3D-printed Ag electrodes for tactile sensing applications, which are used in the tactile sensing array system on a wireless soft robotic gripper [101]. All essential copyrights and permissions received.

The flexible polymer/composite film-based TENG devices have demonstrated substantial potential for the enhancement of electrical output and various self-powered sensing applications, including acoustic, tactile, etc. For example, G.M. Rani et al. successfully fabricated a nature-driven CF-CNT TENG device based on the carbon nanotubes (CNT) decorated on the cigarette filter (CFs) [94]. The assembled CF-CNT-based TENG demonstrated high and stable electrical output, and it employed various commercial portable electronics. Furthermore, the proposed device can be used as a sound-driven TENG that works efficiently across different bandwidth frequencies. These results demonstrate that the CF-CNT TENG has immense potential in various sensing, acoustic, and energy harvesting applications. Also, S.K. Ghosh et al. have developed a high-performance stretchable TENG based on a sandwich structure between the Ag NWs electrodes and hierarchically engineered spongy TPU composite polymer with ferroelectric barium titanate (BTO) and 2D MXene (Ti_3_C_2_T_x_) nanosheets, as shown in Figure 6a, ref. [95]. The Mxene sheets increase the dielectric properties by coupling with BTO particles, resulting in high and stable electrical output from the TENG. The fabricated TENG exhibits a high electrical output power density of 6.65 W/m^2^, high pressure sensitivity, and efficiency. The TENG enables a broad range of real-time applications, including mechanical energy harvesting, vital-sign monitoring, acoustic sensing, and gesture-sensing functionality of a robotic hand. S. Sardana et al. have designed an electrospun triboelectric nanogenerator (TENG) based on the MXenes loaded cellulose acetate NFs, as shown in Figure 6b, ref. [96]. The fabricated TENG generated a high output power density of 1.361 W/m^2^. Further, they assembled an MXene/TiO_2_/C-NFs heterojunction-based sensory component using cellulose nanofibers for the detection of NH_3_. The sensor exhibits excellent reproducibility, high sensitivity, and fast response/recovery time of 76 s/62 s at room temperature. The proposed sensor demonstrated its possibilities as a gas-sensing network driven by human motion energy and external battery dependence for environment monitoring applications. L. Yang et al. have proposed a novel liquid metal (LM)-incorporated thermoplastic polyurethane (TPU) nanofiber-based TENG for mechanical energy harvesting and self-powered sensing properties [97]. Dual optimization of interfacial polarization and charge trapping capabilities was achieved by incorporating the LM nanoparticles into the nanofiber network. The proposed SM-TENG demonstrated the highest electrical output and ultralong-term stability results. This SM-TENG exhibits a high-pressure-sensitive triboelectric response of 6.11 V/kPa, and it enables multimodal sensing properties, including gait analysis, information transmission, physiological signal monitoring, and encrypted human–machine communication. These results are promising for next-generation smart healthcare systems and human–machine interfaces. On the other hand, M. Anithkumar et al. fabricated a novel hybrid nanogenerator (HNG) with multifunctional composite film (MFC), made up of Cu_2_O-doped 0.3Ba_0.7_Ca_0.3_TiO_3_-0.7BaSn_0.12_Ti_0.88_O_3_ nanoparticles (Cu_2_O-BCST NPs) [98]. The HNG demonstrated an outstanding electrical output due to the synergistic properties of the MFC. Further, DL-HNG was assembled with a matrix structure using 3D-printed masks; its demonstrations are systematically operated. The DL-HNG can also be employed as a flexible self-powered trackpad (or touch) sensor to control the mouse pointer in computer or touch-screen display devices. Q. Yi et al. have established a novel 3D printer and integrated a self-powered wearable sensing system for real-time vital sign monitoring applications [99]. The TENG fabricated using Mxene ink is coupled with a styrene–ethylene–butylene–styrene (SEBS) substrate with a positive triboelectric property and high stretchability. The device exhibits excellent power density, high sensitivity of 6.03 kPa^−1^, and a fast response time of 80 ms. The proposed TENG employed as a wearable system for continuous and real-time physiological biosignal monitoring under human motions and signaling exhibits potential in sensing applications. Also, in Figure 6c, Q. Liu et al. have developed a surface-patterned PVDF-HFP/PVC/TiO_2_ composite film-based flexible triboelectric nanogenerator for tactile sensing applications [100]. The prepared composite films show excellent hydrophobic nature with a rough surface, which enables the device to overcome the effect of water molecules on charge transfer in the humid environment, thus resulting in high and stable electrical output from the corresponding TENG device. The proposed TENG demonstrated the mechanical energy harvesting from human movements as well as a self-powered sensor in the tactile sensing applications. In addition, J. Ji et al. have designed a flexible tactile sensor based on the templated laser-induced graphene (TLIG) 3D-printed on Ag electrodes, as shown in Figure 6d, ref. [101]. The designed tactile sensor obtains a high sensitivity of 52,260.2 kPa^−1^, a broad detection range up to 1000 kPa, a quick response and recovery time of 12 and 16 ms, and high stability over 10,000 cycles. The TLIG device acts as a sensory receptor for the soft robotic gripper, and its ability for texture recognition is further demonstrated through the implementation of a soft tactile sensing array. Moreover, M. Lou et al., C. Ning et al., F. Chen et al., W. Xu et al., Y. Yang et al., and Y. Li et al. also designed a flexible polymer/composite film-based TENG device for energy harvesting and real-time acoustic and tactile sensing applications [102,103,104,105,106,107]. From these results, the flexible polymer/composite film-based TENG have been widely used in different practical and real-time scenarios, including energy harvesting and various sensor applications. Table 2 and Table 3 represent the summary of various TENG sensors with their electrical output results, sensing parameters, and applications. Furthermore, the comparison table of the TENG device with various important parameters, including materials, electrical output, stability/durability, cost of fabrication, and their application areas, was summarized in Table 4.

## 9. Conclusions and Feature Perspectives

In conclusion, this review highlights recent progress in the development of flexible triboelectric polymer/composite films for TENGs designed for energy harvesting, wearable technologies, and self-powered sensing applications. Advances in polymer engineering and composite-film preparation show that the addition of selected filler materials can substantially improve dielectric properties, charge-generation ability, mechanical strength, and robust behavior under various environmental conditions. The combined behavior of polymers and engineered nanofillers has enabled flexible TENGs that deliver enhanced electrical output while also providing consistent sensing functions for motion tracking and physiological monitoring. These developments highlight the role of material innovation in affecting the performance constraints of traditional triboelectric films and in expanding the operational versatility of TENG devices. Finally, practical and real-time applications enabled by TENG devices are also introduced, such as wearable, portable, health monitoring, acoustic, tactile, and self-powered pressure sensor systems. These findings show that continuity and multifunctional features can significantly improve and broaden an active device’s capabilities in practical and real-time application scenarios.

With these advances, ongoing innovation has generated a wide range of studies offering practical strategies for the realization of continuous and multimodal active devices, thereby highlighting their strong potential for deployment in diverse real-world applications. Over the past decades, flexible triboelectric polymer/composite films have played a vital role in advancing TENG devices, mainly due to their ability to deliver higher electrical output, mechanical strength, and support for distinct functional parameters. These strengths position polymer or composite film-based TENGs as robust candidates for wearable energy harvesters and self-powered sensor devices, where devices must remain lightweight, flexible, deformable, and reliable during continuous use. However, applying these materials in practical or real-world application scenarios introduces some challenges. Wearable and textile systems require films that can endure repeated bending, stretching, and surface friction without losing electrical performance. Self-powered sensing applications must also ensure that the materials operate stably under different humidity/temperature ranges and long-term cyclic tests.

Moving forward, several research areas merit focused research to accelerate the practical use of these flexible polymers and composite materials. Enhancing electrical output performance remains a priority and can be achieved by designing these composite films by incorporating high dielectric permittivity fillers, strengthening interactions at polymer and filler interfaces, and producing multiscale structures that promote efficient charge generation and movement. Improving mechanical stability and long-term durability is equally important, as composite films can degrade when exposed to moisture, heat, or sustained mechanical stress. Integrating protective surface layers, reinforcing polymer networks, or using materials capable of employing a self-healing nature may help address these concerns. Another promising direction is the development of flexible polymer composite films with built-in sensing functions. By tailoring the composition or integrating responsive nanomaterials, a TENG device could simultaneously harvest energy and monitor mechanical or environmental changes, which would be valuable for advanced wearables, motion tracking, tactile pressure sensing, and health-monitoring technologies. Also, the integration of TENGs with energy storage, wireless communication modules, and multifunctional sensing components will be essential for realizing fully self-powered systems. Overall, continued innovations in polymer/composite material fabrication, structural design, and device integration are expected to accelerate the development of flexible composite film-based TENGs, paving the way for their potential applications in energy harvesting, wearable electronics, self-powered smart sensing networks, and health-care monitoring.

## Figures and Tables

**Table 1:** Comprehensive list of different fabrication processes of polymer/composite films based on TENGs for various TENG applications.

Polymer/ Composite Film	Fabrication Method	Electrical Output			Application	Ref.
		Voltage	Current	Power		
NaYF_4_: Yb,Er@ Au (Ln@Au) nanocomposite	Hydrothermal	424 V	8.5 µA	3.7 W/m^2^	Mechanical energy harvesting	[60]
Pd@ZnO/MoSe_2_@Cu-Ni electrode	Spray coating	200 V	--	17.5 μW	Mechanical energy harvesting and gas sensing	[61]
HWA-PPFC	Plasma treatment	200 V	30 μA	11 W/m^2^	Raindrop energy harvesting	[62]
MN-PDMS	Laser-treated micropattern	73.6 V	36 μA	0.264 W/m^2^	Human motion sensor	[63]
BC/HEC	Vacuum filtration/Dip-coating	76.61 V	8.68 μA	290.7 μW	Mechanical energy harvesting	[64]
CN/GO/ZIF-8/THS	Hydrothermal	184.5 V	22.8 μA	41.32 μW/cm^2^	Wearable and self-powered sensor	[65]
SBT/PGA	Solid-state and drop-casting	~239 V	~7.5 μA	2 W/m^2^	Energy harvesting and self-powered sensor	[59]
PE/PDMS and PA/PEG	3D printing	306 V	6.14 mA	236.67 W/m^3^	Energy harvesting	[66]
PVDF-CuO	Electrospinning	7.5 V	--	--	Biomechanical energy harvesting	[67]
PCA PNMs-TCP HNMs	Electrospinning	49.29 V	--	0.054 W/m^2^	Energy harvesting and self-powered sensor	[68]
PVDF-TrFE/PEDOT:PSS/MAPbI_3_	Spin coating	--	--	12.5 mW/cm^2^	Mechanical energy harvesting	[69]
PDMS/Silica	Blade coating	254 V	20.4 μA	4.37 W/m^2^	Energy harvesting	[70]
PDMS/Ag NW Composite	Casting and Rolling	33.4 V	4.5 mA	0.162 W/m^2^	Mechanical energy harvesting	[71]
PVDF/PVA	Solution casting	230 V	6 μA	3.1 W/m^2^	Self-powered electronic	[72]
MWCNT/PEDOT:PSS@Nylon	Vacuum filtration	109.6 V	--	--	Wearable electronics	[73]
PDMS/Mxene	Spin coating	453 V	131 μA	--	Acoustic and wind energy harvesting	[74]
PVDF/GO@PHBV	Electrospinning	340 V	78 μA	0.141 mW	Energy harvesting	[75]

**Table 2:** A summary of TENGs and various sensor systems, highlighting their sensing parameters, and application fields.

Materials (Polymer/ Composite Film)	Electrical Output (Voltage, Current, and Power)			Sensing Parameters		Application	Ref.
	V	I	PD	Sensitivity	Response Time		
PVDF-HFP/BWO CPF	140	5 µA	43.3 mW/cm^2^	0.88 V·cm^−1^	5 ms	Energy harvesting and sensor	[76]
PVA-NH_2_ FrGO	322.46 V	78.22 µA	192 mW	--	--	Energy harvesting and self-powered sensor	[77]
rGo@PANI@CF	160 V	9 μA	0.576 W/m^2^	--	--	Signal sensing and energy harvesting	[78]
PDMS-BSFO	152 V	10.6 µA	4.71 W/m^2^	--	--	Energy harvesting and sensing	[79]
MCC/PEI/ FeCl_3_	248.3 V	5.3 μA	0.575 W/m^2^	3.136 V/N	60 ms	Energy harvesting and self-powered multi sensor	[80]
GO/PDMS	415 V	5.06 µA	552 µW	0.385 V/N	--	Energy harvesting	[81]
PVA-Natural fibers (NF)	154.64 V	6.29 µA	28.65 mW/m^2^	--	--	Bio-mechanical sensor	[82]
Modified HZC fabric	110 V	18 μA	0.625 W/m^2^		2 ms	Mechanical energy harvesting and multi-sensing applications	[83]
PVDF-HFP/SEBS@ Cs_3_Bi_2_Br_9_	400 V	1.63 µA/cm^2^	2.34 W/m^2^	--	--	Wearable and self-powered sensor	[84]
DA/CNT/Py@ CC	300 V	50 µA	2.6 W/m^2^	--	--	Wearable applications and self-powered sensor	[85]
PTFE-SS @PA66-SS	4 V	26.112 nA	--	2.942 V·kPa^−1^	123 ms	Wearable applications and a self-powered pressure sensor	[86]
PVDF/G-CF	71.5 V	0.36 mA/cm^2^	25.5 mW/m^2^	3.48286 V·kPa^−1^	0.16 s	Wearable sensor, pressure sensor, and human motion monitoring	[87]
Physiological saline/silicone rubber	57.0 V	1.7 mA/m^2^	11.6 W/m^2^	--	--	Wearable applications and a self-powered energy harvester	[88]
Bacterial cellulose/chitosan (BC/CS)	23 V	500 nA	3.25 mW/m^2^	0.24 V·kPa^−1^	--	Mechanical energy harvesting and pressure sensor	[89]
MXene/PU	7.5 V	17 nA	--	2.64819 V·kPa^−1^	140 ms	Pressure sensor	[90]
PP@AgH/PVA	--	--	3.07 W/m^2^	22.86 V/N	--	Wearable self-powered pressure sensing and energy harvesting	[91]
n-Cu/m-PDMS	~13 V	--	~33 µW/cm^2^	0.192 kPa^−1^	8 ms	Self-powered pressure sensing and health monitoring	[92]
ASNb/ecoflex CF	270 V	9 µA	4 W/m^2^	--	--	Mechanical energy harvesting and smart sensing	[93]
CF-CNT	60 V	1.805 µA	110.6 mW/m^2^	--	--	Acoustic energy harvesting	[94]
TPU/BTO/MXene NCF	260 V	160 mA/m^2^	6.65 W/m^2^	4.6 V·kPa^−1^		Energy harvesting, acoustic, and gesture sensing	[95]
MXene/TiO_2_@CNF	140 V	92 μA	~1.36 W/m^2^	--	76 s	NH_3_ gas sensor	[96]
TPU@LM	162 V	3.3 μA	0.176 W/m^2^	6.11 V·kPa^−1^	4.9 s	Pressure sensor and health monitoring	[97]
Cu_2_O-BCST@ PDMS	176.41 V	--	0.168 W/m^2^	--	--	Self-powered touch sensor	[98]
Mxene/SEBS	--	--	~0.81 W/m^2^	~ 6.03 kPa^−1^	~80 ms	Wearable sensor for wireless healthcare monitoring	[99]
PVDF–HFP/PVC/ TiO_2_ CF	235 V	35 μA	1.4 W/m^2^	--	--	Energy harvesting and self-powered tactile sensing	[100]
Templated laser-induced graphene (TLIG)	--	--	--	52,260.2 kPa^−1^	12 ms	Tactile sensor, health monitoring, and texture recognition	[101]
Nylon/PTFE	2.17 V	~22 nA	9.9 μW/m^2^	1.33 V·kPa^−1^	--	Pressure sensor, human motion, and pulse monitoring	[102]
CNT/AgNWs@PDMS	22 V	0.6 µA	21.5 µW/m^2^	5.2 mV·Pa^−1^	--	Pressure and tactile sensing applications	[103]
PVDF/CF	400 V	175 µA	7 W/m^2^	--	--	Self-powered sensor and acoustic energy harvesting	[104]
PVDF/Nylon	124 V	23.5 μA	1.28 W/m^2^	53.6 V·Pa^−1^	--	Pressure sensor and acoustic energy harvester	[105]
PDMS/ITO	−1000 V	8 mA/m^2^	0.5 W/m^2^	~0.29 V·kPa^−1^	--	Pressure and tactile sensor system	[106]
AgNWs/SA	53 V	0.37 μA	4 μW	0.237 V/kPa	100 ms	Pressure and tactile sensor system	[107]

**Table 3:** A summary of TENGs and various sensor systems, highlighting their electrical output performance, energy conversion efficiency, sensitivity, and temperature fields.

Materials (Polymer/ Composite Film)	Electrical Output (Voltage, Current, and Power)			Efficiency	Sensitivity	Temperature Dependency	Ref.
	V	I	PD				
PVC/TiO_2_ NPs	121 V	11.1 μA	141 μW/cm^2^	20%	2.03 V·kPa^−1^	Stable output at 25–55 °C	[108]
P-ZnO@ PVDF/TBAHP TLNM	98 V	18.7 μA	362.5 mW/m^2^	42.8%	--	Stable output at 10–45 °C	[109]
PVDF/MWCNT/BaTiO_3_	48.46 V	1.22 mA·m^−2^	29.27 mW/m^2^	31.62%	--	--	[110]
Liquid metal	679 V	9 μA	6.7 W/m^2^	70.6%	--	--	[111]
PTFE	76 V	0.74 µA	28 µW	29.7%	--	--	[112]
Phosphorus-doped g-C_3_N_4_@PVDF	177 V	15 μA	118 μW/cm^2^	--	1.48 V·kPa^−1^	--	[113]
PVDF/3wt.%PMMA	600 V	1.2 μA	0.750 W/m^2^	--	0.45 V·kPa^−1^	--	[114]
CS/GNPs CFs	166.25 V	13.56 µA	44 mW/m^2^	--	0.96 V·kPa^−1^	40 ± 5 °C	[115]
PVA/rGO NFs	728 V	22 μA	7.2 mW	73%	--	--	[116]
SBT/PGA	239	7.5 μA	2 W/m^2^	48%	--	Almost stable output at 30–60 °C	[59]

**Table 4:** A summary of TENGs and various materials, highlighting their electrical output performance, durability, cost of fabrication, and their applications.

Materials (Polymer/ Composite Film)	Electrical Output (Voltage, Current, and Power)			Durability/Stability	Cost of Fabrication	Applications	Ref.
	V	I	PD				
P(VDF-TrFE)/BaTiO_3_	315 V	6.7 µA/cm^2^	141 μW/cm^2^	10,000 cycles	Moderate cost (electro-spinning)	Wearable electronics and a pressure sensor	[116]
PVA/rGO NF@PTFE/PI	728 V	22 µA	~2.25 W/m^2^	Not reported	Moderate cost (electro-spinning)	Energy harvesting for self-charging supercapacitor	[117]
Corn husk-based TENG	630 V	0.79 mA	32.75 mW/ cm^2^	Stable output ~7200 cycles	Very low (bio-materials)	Powering electronic devices	[118]
PA-66 fabric/ PP fabric	210 V	28.3 μA	0.901 W/m^2^	Stable output ~6000 cycles	Low cost	Self-powered pedestrian volume and pugilism training monitor	[119]
Gelatin/polyimide@ PANI/NiCo_2_O_4_	400 V	49 μA	0.246 W/m^2^	10,000 cycles	Low cost	Mechanical energy harvesting and gas sensor	[120]
MoS_2_/CNT@ Nylon	300 V	11.5 μA	0.134 W/m^2^	Stable 3000 cycles (6 months)	Moderate cost (electro-spinning)	Mechanical energy harvesting	[121]
TOCN/CCTO composite aerogel	152 V	33.8 μA	0.483 W/m^2^	Stable ~50,000 cycles	Low-cost	Energy harvesting for electronic gadgets	[122]
SrTiO_3_@PDMS Composite sponge	338 V	9.06 μA/cm^2^	6.47 W/m^2^	Stable ~15,000 cycles	Low-cost (blade coating)	Energy harvesting for electronic gadgets	[123]
PCL/CNTs	808 V	23 μA	54 W/m^2^	Stable ~10,000 cycles (21 days)	Moderate cost (electro-spinning)	Mechanical energy harvesting and sensing	[124]
PTFE balls/Al	--	20.91 μA	34.65 W/m^3^	--	High cost (complex design)	Harvesting wave energy, sensors, and robotics	[125]

## Data Availability

Data will be made available on request.
